# DDX21 at the Nexus of RNA Metabolism, Cancer Oncogenesis, and Host–Virus Crosstalk: Decoding Its Biomarker Potential and Therapeutic Implications

**DOI:** 10.3390/ijms252413581

**Published:** 2024-12-19

**Authors:** Yalan Xiao, Jiankun Fan, Zhigang Li, Yu Hou

**Affiliations:** 1 Department of Radiological Medicine, School of Basic Medical Sciences, Chongqing Medical University, Chongqing 400016, China; xiao_yalan@stu.cqmu.edu.cn (Y.X.); 2021130115@stu.cqmu.edu.cn (J.F.); 2 Chongqing Key Laboratory of Hematology and Microenvironment, Chongqing Medical University, Chongqing 400016, China

**Keywords:** DDX21, RNA metabolism, cancer biomarker, viral infection

## Abstract

DDX21, a member of the DEAD-box RNA helicase family, plays a pivotal role in various aspects of RNA metabolism, including ribosomal RNA (rRNA) processing, transcription, and translation. Its diverse functions in cancer progression and viral infections have attracted considerable attention. DDX21 exerts a pivotal function through ribosomal DNA (rDNA) transcription and rRNA processing. DDX21 is involved in different biological processes of mRNA transcription. It interacts with transcription factors, modulates RNA polymerase II elongation, binds R-loops to regulate transcription, and participates in alternative splicing. The elevated expression of DDX21 has been observed in most cancers, where it influences tumorigenesis by affecting ribosome biogenesis, transcription, genome stability, and cell cycle regulation. Additionally, DDX21 plays a key role in the antiviral defense of host by interacting with viral proteins to regulate essential stages of the infection process. This review provides a thorough examination of the biological functions of DDX21, its involvement in cancer progression and viral infections, and its potential as both a biomarker and a therapeutic target. Future studies should aim to clarify the specific mechanisms of the activity of DDX21, advance the development of targeted therapies, and assess its clinical relevance across various cancer types and stages.

## 1. Introduction

The DEAD-box RNA helicase family, a subset of the helicase superfamily II, is one of the largest helicases families found in eukaryotes, archaea, and bacteria [1]. Characterized by the conserved Asp-Glu-Ala-Asp (DEAD) motif, this family includes 26 members in *Saccharomyces cerevisiae* and 37 members in *Homo sapiens* [2,3]. These helicases possess nine conserved motifs responsible for ATP binding and hydrolysis, RNA binding, and ATP-dependent RNA remodeling, which drive their roles in RNA metabolism, such as transcriptional regulation and translation initiation [4,5,6]. Importantly, these functions are vital for maintaining cell survival. Beyond RNA metabolism, DEAD-box RNA helicases participate in diverse cellular processes, including embryonic development, cell proliferation, hematopoiesis, innate immunity, and immune regulation [5,7,8]. Their involvement extends to pathological contexts such as cancer progression and inflammatory responses, underscoring their pivotal roles in both physiological and pathological mechanisms.

Currently, studies on members of the DEAD-box helicase family are rapidly expanding, with DDX21 (also known as RH-II/Guα) drawing particular attention [9]. DDX21 is deeply involved in RNA metabolism and represents a promising molecular target for therapeutic interventions. Understanding its structural complexities and functional roles is crucial for elucidating its diverse cellular functions. Recent research has increasingly highlighted the importance of DDX21 in cancer and viral infections, revealing its potential as a biomarker and therapeutic target in these diseases [10,11,12,13,14]. Although existing reviews have focused on its role in gene expression regulation in diseases, few have conducted an in-depth analysis of its structural characteristics, functional properties, and broader therapeutic applications from a holistic perspective. This review aims to provide a detailed analysis of DDX21, including its structural and functional features, its roles in cancer therapy and viral infection management, and its potential as a therapeutic target, with an emphasis on RNA metabolism, oncology, and host–pathogen interactions.

## 2. The Structure of DDX21

DDX21, also known as RH-II/Guα, is a member of the DEAD-box helicase family. Human DDX21 consists of 783 amino acids (87 kDa) and primarily localizes to the nucleolus. This helicase exhibits ATP-dependent helicase activity for unwinding double-stranded RNA (dsRNA) and RNA folding activity for single-stranded RNA (ssRNA), governed by distinct structural domains [15,16]. Key factors such as NTP, Mg^2+^, and mutations significantly influence its helicase activities [15]. These functions underscore the essential role of DDX21 in correcting RNA misfolding, facilitating proper folding, and supporting critical biological processes [17].

**Figure 1:**
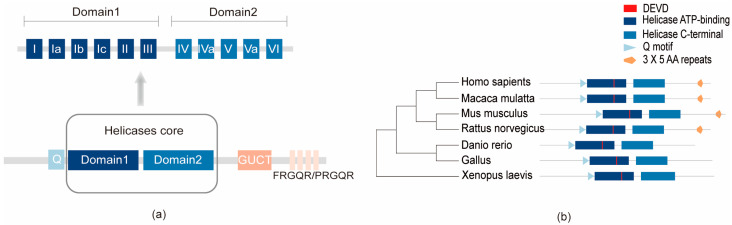
The structure and phylogenetic tree of human DDX21: (**a**) The specific structure of human DDX21. (**b**) The phylogenetic tree of DDX21. Amino acid alignment of *Homo sapiens* (Q9NR30), *Macaca mulatta* (H9G1S2), *Mus musculus* (Q9JIK5), *Rattus norvegicus* (Q3B8Q1), *Danio rerio* (A0A2R9YJN8), *Gallus* (A0A8V0ZUQ0), and *Xenopus laevis* (Q9DF36). Specific structural domains include Q motif, Helicase ATP-binding, DEVD box, Helicase C-terminal, and 3 × 5 AA repeats.

Human DDX21 is composed of a central helicase domain, an arginine-rich N-terminal region, and a glycine/arginine-rich C-terminal region, all of which are crucial for cell growth and cell cycle progression [17]. The central helicase domain, containing two highly conserved RecA-like subdomains (domains 1 and 2), harbors 12 motifs critical for ATP binding and hydrolysis (motifs Q, I, II/DEVD, VI), RNA binding (motifs Ia, Ib, Ic, IV, IVa, V) and communication between ATP-binding and RNA-binding sites (motif III, Va) [18,19]. The C-terminal domain (residues 749–810), with three FRGQR repeats and one PRGQR repeat, is responsible for RNA folding activity, while its GUCT domain remains functionally unclear [16,20] (Figure 1a). Nuclear localization signals at both termini enable its nuclear import. Notably, recent studies reveal that the C-terminal domain interacts with viral proteins, impacting viral infection progression [13,21].

Human DDX21 function s as a homodimer, with dimerization mediated by a hydrophobic domain within residues 570–620. This dimeric structure is essential for ATP-dependent dsRNA unwinding and ATP-independent G-quadruplex remodeling [19]. DDX21 shares its homology with its paralog DDX50 (RH-II/Guβ), a product of gene duplication. Unlike DDX21, DDX50 lacks helicase functionality, as it undergoes alternative splicing and contains a pseudogene [22]. Phylogenetic analysis across vertebrates, including human, rhesus monkey, mouse, and zebrafish, reveals highly conserved domains such as the Q motif, the Helicase ATP-binding domain, DEVD-box, and the Helicase C-terminal domain. Interestingly, a repetitive sequence at the C-terminus is unique to mammals like humans and rodents, suggesting species-specific adaptations (Figure 1b). Phylogenetic analysis highlights a closer relationship between humans and rhesus monkeys, providing insights into the evolutionary conservation of the structure and functions of DDX21.

## 3. DDX21 in RNA Metabolism

**Figure 2:**
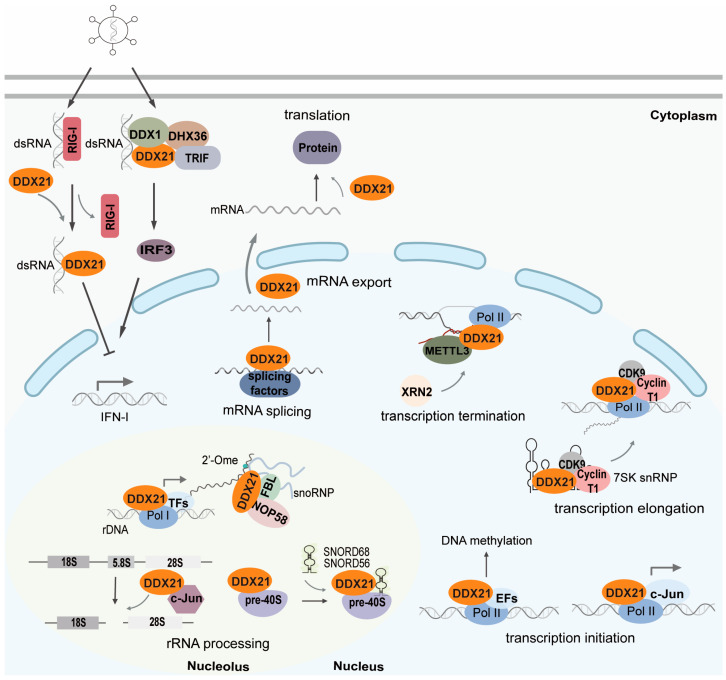
DDX21 in RNA metabolism. In the nucleolus, DDX21 participates in ribosomal RNA (rRNA) synthesis and processing. It orchestrates transcriptional initiation in the nucleus by co-transcribing with transcription factors or recruiting epigenetic modifiers. DDX21 binds to the 7SK small nuclear ribonucleoprotein (7SK snRNP) and facilitates the release of the positive transcription elongation factor b, which promotes transcriptional elongation. Additionally, DDX21 associate with R-loops and recruit the methyltransferase METTL3 to install m6A modifications co-transcriptionally at the transcriptional end sites, subsequently recruiting XRN2 for transcription termination. DDX21 also plays a role in alternative splicing and regulates the nuclear export and translation of viral mRNA. Furthermore, DDX21 acts as a sensor for double-stranded RNA (dsRNA) and modulates innate immune responses.

The unique structure of DDX21, including its specific domains, plays a critical role in determining its distinct functions. Its subcellular localization is a key factor influencing these functions, particularly its dynamic movement from the nucleolus to the nucleoplasm and other cellular compartments. As an RNA helicase, DDX21 is essential for various aspects of RNA metabolism, including ribosomal RNA (rRNA) processing, transcription, splicing, RNA nuclear export, and translation (Figure 2).

### 3.1. rRNA Processing

As a nucleolar protein, DDX21 participates in the transcription, processing, and modification of rRNA, facilitating the assembly and maturation of ribosomal subunits [23,24,25]. DDX21 enhances ribosomal DNA (rDNA) transcription by binding to the rDNA promoter region. However, the precise nature of DDX21’s direct interaction with rDNA remains unclear [24]. A long non-coding RNA (lncRNA) known as SLERT, which contains small nucleolar RNA (snoRNA) at its 3′ end, binds to DDX21 and modulates its conformation, releasing its inhibition of RNA polymerase I (Pol I). This enables Pol I to bind to rDNA and enhance pre-rRNA transcription [26]. Recent studies also show that calmodulin interacts with DDX21 in a calcium-dependent manner, altering its conformation and facilitating the release of the sequestered Pol I catalytic subunit RPA194, thereby promoting rDNA transcription [27]. These findings highlight potential conflicts in DDX21’s regulatory role in rDNA transcription. While DDX21 enhances rDNA transcription by binding to its promoter, it also requires conformational changes mediated by other proteins or RNAs to alleviate its inhibition of Pol I. The precise balance and interplay between these roles remain unclear, necessitating further investigation to address these apparent contradictions.

The modification of rRNA, a critical step in its maturation, is primarily guided by small nucleolar ribonucleoproteins and can occur co-transcriptionally [28]. DDX21 is involved in both stages, particularly by interacting with C/D box snoRNAs to facilitate rRNA modifications, including 2′-O methylation of 18S rRNA and 28S rRNA [29,30]. Notably, DDX21 is essential for recruiting late-acting snoRNAs, such as SNORD56 and SNORD68, to modify pre-40S rRNA during its processing. In contrast, early-acting snoRNAs are not affected by DDX21 depletion [31]. Despite these insights, the exact mechanisms by which DDX21 influences ribosome biogenesis remain an area requiring further investigation.

### 3.2. Transcription

DDX21 is involved in the regulation of gene transcription at every stage, ranging from the precise initiation to efficient RNA synthesis during elongation and proper pausing during termination [24,32,33]. It coordinates with RNA polymerase, transcription factors, and other regulatory proteins to ensure the spatiotemporal specificity of gene expression.

During transcription initiation, DDX21 recruits epigenetic modifiers to promoter regions, influencing histone modifications and promoting transcriptional activation [24]. For example, in human colorectal cancer (CRC) cells, DDX21 binds to the *CDK1* promoter, interacting with WDR5 to enhance H3K4me3, which activates *CDK1* expression and supports cell proliferation [34]. Conversely, in human breast cancer cell lines, DDX21 also participates in transcriptional repression at the *SNAIL* promoter by recruiting SUZ12 from the PRC2 complex to induce histone H3K27 trimethylation (H3K27me3), suppressing *SNAIL* expression [35]. This dual functionality emphasizes DDX21’s ability to interact with distinct epigenetic modifiers and selectively modulate chromatin states based on specific promoter contexts. Furthermore, in mammalian cells, DDX21 directly interacts with c-Jun to modulate AP-1 activity and increase the mRNA of cell cycle-related genes like *cyclin D1* [36,37]. These studies further emphasize the multifunctionality of DDX21 in transcriptional regulation.

Once transcription has begun, the efficiency of RNA polymerase II (Pol II) elongation determines the integrity of RNA synthesis. DDX21 regulates elongation through its ATP-dependent helicase activity. It facilitates the positive transcription elongation factor b release from the 7SK small nuclear ribonucleoprotein complex, promoting transcriptional elongation in human cells [24,32]. DDX21 also resolves R-loops formed during transcription, preventing RNA polymerase stalling and ensuring genome stability in breast cancer cells [38].

Finally, DDX21 assists in the termination process by resolving R-loops and recruiting the METTL3 to add m6A modifications to nascent transcripts in human cells. These modifications mark the start of RNA maturation and facilitate XRN2 recruitment, which further aids RNA polymerase release [33,39,40]. This coordination ensures successful transcription termination while maintaining genome stability.

### 3.3. Splicing

Splicing is a crucial step in mRNA maturation within RNA metabolism, playing a vital role in regulating gene expression by modulating mRNA diversity, stability, and function [41]. In HeLa, DDX21 regulates alternative splicing by upregulating AGO2 and participates in the selective splicing of the *SMN2* gene through an indirect interaction with AGO2. This interaction leads to the generation of distinct *SMN2* transcripts with varying expression [42]. However, the precise mechanism behind this interaction remains unclear. In addition, DDX21 has been found to localize to specific SCUGSDGC motifs within mRNA introns in a glucose-dependent manner, where it promotes the splicing of key pro-differentiation genes [43,44]. This suggests that DDX21 may function not solely as a helicase but through this unique localization to regulate splicing. These findings provide an initial understanding of DDX21’s role in alternative splicing, though further studies are needed to clarify whether DDX21 directly regulates this process and to uncover the underlying mechanisms.

### 3.4. RNA Nuclear Export

RNA nuclear export is a critical step in RNA metabolism, regulating the cytoplasmic localization of RNA and its subsequent translation. Although research on DDX21’s role in mRNA nuclear export is limited, one study has demonstrated its involvement in regulating viral RNA export. Specifically, DDX21 enhances Rev-dependent RNA nuclear export, facilitating the transport of unspliced or partially spliced HIV-1 RNA from the nucleus to the cytoplasm [45]. This finding not only highlights DDX21’s role in viral RNA export but also suggests that it may have broader functions in RNA nuclear export, potentially extending to host RNA as well. This insight provides a valuable foundation for future studies exploring DDX21’s involvement in nuclear export processes.

### 3.5. Translation

In eukaryotes, most cellular genes are transcribed as monocistronic mRNAs, whereas many animal viruses produce polycistronic mRNAs to efficiently express multiple proteins from a single transcript [46]. Two main reinitiation mechanisms are observed in both eukaryotic and viral mRNAs: one involves mRNAs with short upstream open reading frames (uORFs) preceding the main coding sequence, while the other involves longer 5′ ORFs, which often encode functional proteins [46,47,48,49]. An example of the latter is the shortest transcript of the Borna disease virus, X/P mRNA, which contains three ORFs: uORF, X, and P [50]. Notably, the termination codon of the uORF overlaps with the initiation codon of ORF X, classifying this mRNA as belonging to the second type. DDX21, a host protein, is phosphorylated and binds to the 5′ untranslated region (UTR) of X/P mRNA, altering its conformation and inhibiting the translation of ORF X. When protein P is expressed, it leads to the dephosphorylation of DDX21, reducing its binding affinity for the 5′ UTR and enabling ribosomal reinitiation at ORF X, thereby enhancing its translation. This represents the first example of self-regulated translational control in a multicistronic mRNA, mediated by both a viral-encoded protein and a host RNA helicase [50]. Although research on the regulatory role of DDX21 in translation remains limited, understanding its involvement in viral translation offers valuable insights into its potential role in regulating eukaryotic translational processes.

## 4. DDX21 in Cancer Oncogenesis

Alterations in DDX21 regulation have been linked to various types of cancer, highlighting its potential as both a biomarker and a therapeutic target. DDX21 is often overexpressed in several cancers, where it contributes to tumorigenesis by affecting key biological processes such as ribosome biogenesis, transcription, genome stability, and cell cycle regulation [25,51]. This section reviews the roles of DDX21 in cancer development, focusing on its involvement in breast cancer, colorectal cancer, gastric cancer, hematologic malignancies, and neurological disorders.

### 4.1. Breast Cancer

Breast cancer is the most common malignancy among women and ranks among the top three most prevalent cancers worldwide [52]. Research on DDX21 in breast cancer has primarily focused on its role in ribosome biogenesis and transcriptional regulation. PARP-1, a key regulator in DNA repair, also binds to snoRNAs and enhances its catalytic activity in nucleosomes independently of DNA damage. Upon activation, PARP-1 ADP-ribosylates DDX21, promoting rDNA transcription. The inhibition of PARP or mutations at the ADP-ribosylation site reduce DDX21 localization to nucleosomes, leading to decreased rDNA transcription, impaired ribosome biogenesis, and reduced cell growth [25]. Additionally, DDX21 interacts with EZH2 and SUZ12 at the *SNAIL* promoter to enhance H3K27me3, epigenetically repressing *SNAIL* transcription and thereby inhibiting epithelial–mesenchymal transition (EMT) and suppressing breast cancer cell invasion and metastasis [35]. Nuclear DDX21 also interacts with c-Jun, a component of the AP-1 transcription factor complex. The phosphorylation of c-Jun at Ser73 is essential for DDX21 to promote AP-1 activity and enhance rRNA processing in multiple breast cancer cell lines [37].

### 4.2. Colorectal Cancer

According to the 2022 global cancer statistics, colorectal cancer is the third most common cancer in terms of incidence and the second leading cause of cancer-related deaths worldwide [53]. Identifying potential target genes is crucial for the precise management of colorectal cancer. Studies have shown that DDX21 is overexpressed in colorectal cancer and plays a significant role in its development [34,51,54]. The upregulation of DDX21 primarily affects genomic stability and cell cycle regulation. Elevated DDX21 impair DNA repair by inhibiting homologous recombination and increasing replication stress, resulting in genomic instability and tumorigenesis [51]. Notably, the genome instability induced by RNA helicases confers alternative sensitivity to chemotherapy, offering insights into precision treatments for RNA helicase-driven cancers. Furthermore, the overexpression of DDX21 has been shown to activate *CDK1* expression by recruiting WDR5 to the *CDK1* promoter or by directly targeting *CDC5L* to modulate its expression. Both mechanisms contribute to colorectal cancer progression by regulating cell cycle dynamics [34,54].

### 4.3. Gastric Cancer

Gastric cancer is a leading cause of cancer-related deaths worldwide. LncRNAs have emerged as potential functional molecules for cancer diagnosis and treatment, with DDX21 identified as a downstream target of several lncRNAs involved in gastric carcinogenesis. For example, lncRNA HCP5 directly targets DDX21, and its downregulation inhibits DDX21 expression in gastric cancer cells [55]. LINC00240 promotes DDX21 stabilization and gastric carcinogenesis by enhancing the interaction between DDX21 and the deubiquitinating enzyme USP10 [56]. Furthermore, lncPLCB1 interacts with DDX21, and the low expression of lncPLCB1 stabilizes DDX21 by reducing its degradation through the autophagosome–lysosome pathway, without affecting its mRNA. The stabilized DDX21 then promotes the upregulation of molecules associated with proliferation and EMT, thereby facilitating gastric cancer development [10]. Additionally, DDX21 directly enhances gastric cancer progression by increasing the mRNA and protein of *cyclin D1* and *CDK2* [57].

### 4.4. Other Cancers

DDX21 has also been studied in other cancers, including neuroblastoma, acute myeloid leukemia (AML), and lymphoma. In these cancers, DDX21 regulates tumor development primarily through its effects on transcription and ribosome biogenesis. In neuroblastoma, where the amplification of the *MYCN* oncogene is commonly associated with poor prognosis, N-MYC upregulates *DDX21* transcription. DDX21 then binds to the promoter region of *CEP55*, promoting the phosphorylation of Pol II and its binding to the transcriptional termination site [58,59]. This enhances transcriptional elongation and, ultimately, neuroblastoma cell proliferation. In AML, the most common type of adult leukemia, relapse and drug resistance are major challenges to patient survival. m6A-binding proteins play a critical role in AML, with IGF2BP2 and IGF2BP3 stabilizing *DDX21* mRNA in an m6A-dependent manner. DDX21, in turn, triggers autophagy by recruiting the transcription factor *YBX1*, which promotes the expression of *ULK1* and enhances AML cell proliferation [60]. Furthermore, in AML1-ETO-driven leukemogenesis, a genome-associated amino-terminal-splitting enhancer induces snoRNA/RNP formation through interaction with DDX21 [29]. In lymphoma, DDX21 also regulates lymphatic endothelial cell responses to Vegfc-Flt4 signaling by balancing ribosome biogenesis and *p53* activity [61]. This balance promotes endothelial cell proliferation and could be targeted in conditions characterized by excessive lymphangiogenesis, such as tumor metastasis or lymphatic malformations.

## 5. DDX21 in Viral Infections

In addition to its well-established role in cancer progression, DDX21 is crucial for the regulation of viral infections, owing to its RNA-binding and helicase activities. This section explores how DDX21 modulates the viral infection process, emphasizing its dual role in both antiviral defense and viral pathogenesis (Table 1).

### 5.1. Innate Immunity

The innate immune system serves as the primary defense mechanism of the host, detecting pathogen invasion through pattern recognition receptors that recognize pathogen-associated molecular patterns (PAMPs) [71,72]. A growing body of research underscores the crucial role of DDX21 in viral processes. Like other helicases, DDX21 contributes to innate immunity by modulating the Interferon beta (IFN-β) signaling pathway, thereby influencing the host’s immune response (Figure 2). DDX21 can interact with other helicases to initiate innate immune reactions during viral infections [62,73]. For instance, research has demonstrated that dsRNA directly binds to helicases such as DDX1, DHX36, and TRIF, triggering downstream interferon responses. In collaboration with DDX1 and DHX36, DDX21 interacts with the adaptor molecule TRIF to recognize viral dsRNA. This interaction activates the NF-κB pathway and induces a type I interferon response in dendritic cells [73]. In another study, caspase-3/6 cleavage of DDX21 at position D126 promotes its translocation from the nucleus to the cytoplasm during viral infection. In the cytoplasm, the cleaved form of DDX21 negatively regulates the IFN-β signaling pathway by disrupting the formation of the DDX1-DDX21-DHX36 complex [74]. Additionally, the collaboration between DDX21 and DDX60 has been shown to enhance the interferon response and demonstrate antiviral properties against the Hantaan virus [62].

The upregulation of DDX21 expression has also been observed during infections with Chum salmon reovirus in the ZF4 cell line and Spring viremia of carp virus in carp. This upregulation leads to the increased production of type I interferons and may contribute to the antiviral immune response [63]. The interaction between the Omicron N protein and human DDX21 has been suggested to enhance the production of the anti-inflammatory cytokine IL-4, potentially alleviating the severity of Omicron infection [64]. In particular, DDX21 contributes to immune responses that are regulated independently of PAMPs. During Influenza A virus infection, DDX21 binds to TRIF to promote the expression and secretion of S100A9 protein into the extracellular space. Extracellular S100A9 triggers damage-associated molecular patterns (DAMPs), promoting inflammation and apoptosis through the TLR4-MyD88 pathway [65]. However, a study also demonstrated that DDX21 negatively regulates natural immunity. In Sendai virus infection, DDX21 competes with retinoic acid-inducible gene I (RIG-I) for binding to dsRNA in the 217–784 amino acid region, inhibiting the RIG-I-mediated production of IFN-β. This inhibitory effect of DDX21 on IFN-β production is independent of its ATPase, RNA helicase, and RNA folding enzyme activities [75]. During Porcine reproductive and respiratory syndrome virus (PRRSV) infection, DDX21 translocated to the cytoplasm, stabilizing the expression of PRRSV nsp1α, nsp1β, and N proteins, thereby inhibiting the host’s innate immune response [14]. These findings reveal the dual regulatory role of DDX21 in innate immunity, demonstrating that it can both enhance and inhibit immune responses depending on the viral and host context. This dual regulatory mechanism likely reflects the complex adaptive strategies employed by the host in response to different viral infections. Future research needs to further elucidate the specific regulatory mechanisms of DDX21 in different viral infections to better understand its role in innate immunity.

### 5.2. Regulating Viral Replication and Transcription

DDX21 is present not only in antiviral reactions but also in the lifecycle of viruses, contributing to their production. In the case of the DNA virus Human cytomegalovirus, it was found that knocking out DDX21 promoted the accumulation of R-loops, which interfered with transcription and prevented the expression of late viral genes necessary for viral replication [67]. DDX21 facilitates viral proliferation through several biological processes, including replication, transcription, protein–protein interactions, and by promoting the assembly of viral particles. The ^763^GSRSNRFQNK^772^ residues at the C-terminal domain of DDX21 are critical for binding to the nucleolar localization signal of the Porcine circovirus (PCV4) Cap protein, targeting the PCV4 Cap to the nucleolus and aiding in viral replication in the cell nucleus [11,21]. DDX21’s role in viral replication extends beyond DNA viruses, as seen in Human immunodeficiency virus (HIV) infection. In this context, DDX21 binds to the HIV Rev protein, a key regulator of the viral life cycle, to modulate gene transcription late in infection. DDX21 interacts with HIV Rev to regulate HIV production by reducing *p24* [18,68]. Additionally, DDX21, in conjunction with other RNA helicases such as DDX1, DDX3, DDX5, DDX17, and DDX56, enhances the function of HIV-1 Rev by stabilizing its binding to viral RNA and promoting the export of viral transcripts to the cytoplasm [45,76]. This dual capacity of DDX21, to both combat and facilitate viral activities, highlights its pivotal role in the complex interplay between host cellular machinery and viral pathogens.

### 5.3. The Dynamic Antiviral and Evasion Mechanisms

Viruses are adept at evading host defenses, and in the early stages of infection, key host genes work to suppress viral activity. However, as the infection progresses, viruses employ various strategies to overcome these host defenses and expand their replication. In the early stages of infection, DDX21 inhibits virus production. As the infection progresses, the virus counteracts this inhibition to facilitate its replication. Researchers have found that in the early stages of Influenza A infection, DDX21 binds to the viral polymerase PB1, which catalyzes RNA biosynthesis, inhibiting Influenza A replication by affecting polymerase assembly. However, in the later stages of infection, the viral NS protein binds to the N-terminal domain of DDX21, displacing PB1 and overcoming the inhibitory effect [66]. Similarly, in cells infected with Dengue virus (DENV), DDX21 translocated from the nucleus to the cytoplasm to activate the innate immune response, thereby inhibiting DENV replication. Subsequently, DDX21 is degraded by the NS2B-NS3 protease complex, disrupting the innate immune response and promoting DENV replication [69]. In Foot-and-mouth disease virus (FMDV) infection, the overexpression of DDX21 inhibits virus IRES-dependent translation and replication. The virus adapts by counteracting host proteins that threaten its replication. FMDV and its non-structural proteins 2B, 2C, and 3Cpro degrade DDX21 through different pathways. FMDV proteins 2B and 2C facilitate DDX21 degradation via the caspase pathway, while 3Cpro mediates its degradation through the lysosomal pathway, counteracting the host’s antiviral response [77]. A similar phenomenon has been observed in Senecavirus A, where DDX21 acts as a potent antiviral factor, but the virus circumvents this defense by orchestrating the degradation of DDX21 [70]. These findings highlight the dynamic regulatory role of DDX21 in innate immunity and viral infections. This dynamic regulation not only reflects the complex interplay between the host and the virus but also reveals how the host employs different mechanisms to inhibit viral replication at various stages of infection.

## 6. DDX21: A Biomarker and Therapeutic Target

In this article, we discussed the regulatory role of DDX21 in RNA metabolism, cancer, and viral infections, summarizing its molecular functions in specific cancers and viral infections. Through this exploration, we found that DDX21 has emerged as a potential biomarker and therapeutic target for various cancers and viral infections, with significant implications for cancer prognosis and antiviral treatment strategies. In the following section, we will delve into the specific research on DDX21 as a biomarker and therapeutic target, as well as its limitations. Given its involvement in these critical processes, DDX21 presents a promising candidate for therapeutic targeting. A deeper understanding of its functions and interactions with RNA and other proteins could open new avenues for drug development and treatment strategies.

### 6.1. DDX21 as a Biomarker and Therapeutic Target in Cancer

Extensive research has focused on the role of DDX21 in breast and colorectal cancers. In breast cancer, DDX21 has been identified as a marker for a subset of patients with high proliferative potential and as a potential therapeutic target [37]. Additionally, its association with early mortality and reduced disease-free survival has been noted [78]. Furthermore, the identification of circDDX21 as a prognostic indicator for triple-negative breast cancer highlights its potential as both a diagnostic biomarker and a therapeutic target for this specific subtype [12]. In CRC, DDX21 has been recognized as a biomarker through bioinformatic analyses, which show elevated expression in cancerous tissues compared to normal tissue [79,80]. ZFAS1, which exerts an oncogenic function, is significantly upregulated alongside increased expression of *DDX21* and *POLR1B* in CRC cells and tissues. This has led to the proposal that the ZFAS1/DDX21/POLR1B signaling axis could serve as a novel biomarker and target for CRC treatment and prognostic evaluation [81]. Beyond breast and colorectal cancers, the significance of DDX21 extends across a broad spectrum of neoplastic disorders. For instance, DDX21 has been recognized as an early diagnostic marker for prostate adenocarcinoma [82]. In gastric cancer, DDX21 has been shown to drive cancer cell proliferation, primarily through the activation of the cyclin D1 and CDK2 signaling pathways, suggesting that DDX21 could serve as a therapeutic target for gastric cancer [57]. Additionally, DDX21 has been implicated as a prognostic factor in osteosarcoma and may represent a potential target for treatment [83]. These findings highlight the potential of DDX21 as a viable target for cancer therapy. However, the development of precise small molecule inhibitors targeting DDX21 is still in its early stages, warranting further investigation in future studies.

The elevated expression of DDX21 in these malignancies underscores its utility as a diagnostic marker and a prospective therapeutic target, while also paving the way for innovative treatment paradigms focused on DDX21. Research has found that DDX21 regulates the cell cycle in CRC by recruiting WDR5 or interacting with CDC5L [34,54]. This suggests that targeting DDX21 directly could be a strategy for treating CRC. Furthermore, targeting proteins that interact with DDX21 or regulating these interactions such as using WDR5 inhibitors or blocking the DDX21-WDR5-CDC5L interaction offers additional possibilities for CRC treatment. However, these approaches require further validation through experimental studies and clinical trials.

Interestingly, studies have revealed that elevated DDX21 protein correlate with longer survival rates in early-stage CRC patients, particularly those with microsatellite instability (MSI), who experience extended disease-free survival. However, no such correlation was found in patients with microsatellite stable subtypes or advanced CRC. The high expression of DDX21 is an independent favorable prognostic marker for early-stage MSI CRC, where higher DDX21 expression is associated with longer disease-free survival [84]. As a nuclear autoantigen, DDX21 may induce DDX21-specific autoimmunity against tumor cells in MSI tumors, which could lead to better clinical outcomes for patients and potentially improve responses to immune checkpoint inhibitors. Identifying DDX21 and other autoantigen markers in cancer tissues may provide a basis for developing more specific cancer immunotherapy strategies in the future.

### 6.2. Therapeutic Strategies Targeting DDX21 in Viral Infections

A promising strategy for developing new antiviral drugs is to identify essential host proteins that viruses rely on for replication and target these proteins or their interactions with viral factors. Given DDX21’s role in modulating innate immunity against viruses, a deeper understanding of its mechanistic functions could lead to the development of novel immunotherapies. Current research is heavily focused on investigating DDX21’s role in viral infections, particularly its ability to inhibit viral expression during the early stages of infection. As viruses evolve to resist the action of DDX21, the critical importance of host proteins in regulating viral activity becomes increasingly evident. This underscores the potential value of targeting DDX21 itself as part of novel antiviral treatment strategies. DDX21 has been identified as a broad-spectrum antagonist against RNA viruses, providing a unique opportunity to develop antiviral medications focused on DDX21 or its interacting proteins [77]. One approach to inhibiting viral replication involves targeting viral proteins such as NS1. Researchers have explored strategies like inducing mutations in critical binding interfaces between NS1 and DDX21 or developing small molecule inhibitors that enhance the binding affinity for this interaction [66]. While these methods have shown promise, there is still a lack of targeted research aimed specifically at developing antiviral therapeutics centered around DDX21. It is therefore recommended that future studies focus on innovative antiviral treatment strategies that target DDX21, helping to broaden the range of antiviral options available.

Building on existing research, new strategies for inhibiting DDX21 function can be developed, particularly those that address challenges related to cell and tissue specificity. Approaches such as nanotechnology or targeted drug delivery systems hold promise for overcoming these issues. Additionally, combining DDX21 inhibition with other treatment modalities, such as immunotherapy or targeted therapies aimed at the tumor microenvironment, could enhance therapeutic efficacy. Ultimately, the identification of DDX21 as a promising biomarker across various cancer types offers significant potential for its use in disease detection, early intervention, prevention, and treatment strategies.

## 7. Conclusions

DDX21, an RNA helicase, plays a crucial role in various developmental and physiological processes. It is primarily localized in the nucleolus, although it is also found in the nucleoplasm. While the domains of DDX21 have been extensively studied, the functional role of the GUCT domain remains poorly understood. As an RNA helicase, DDX21 is essential for RNA metabolism, including processes such as rRNA processing, transcription, splicing, RNA nuclear export, and translation. However, its involvement in other RNA metabolic processes is still not fully elucidated. For instance, the role of DDX21 in regulating non-coding RNA, including its structural basis and downstream effects, requires further investigation. Currently, DDX21 is upregulated in most tumors, where it contributes to tumorigenesis through processes such as transcriptional regulation and ribosome biogenesis. However, the mechanisms behind its paradoxical roles as both an oncogene and a tumor suppressor remain incompletely understood. DDX21 exhibits diverse and context-dependent roles across cancer types, acting as both a promoter of tumor progression through genomic instability and cell cycle regulation and a suppressor of metastasis via the epigenetic repression of EMT-related genes. These dual functions highlight potential conflicts in its mechanistic impact, which varies based on cellular context and upstream regulators. Additionally, while DDX21-driven genomic instability may enhance chemosensitivity in some cancers, its role in promoting autophagy and survival in others suggests opposing therapeutic implications, underscoring the need for precise, cancer-specific targeting strategies. In viral infections, DDX21 not only modulates viral replication but also plays a role in the host’s innate immune response, exerting both positive and negative effects. Interestingly, the regulation of host DDX21 is dynamic during different stages of viral infection. Further studies are needed to resolve these issues. As a complex molecular target, DDX21 warrants further research to elucidate its specific mechanisms of action in different cancer types. By exploring its functions and mechanisms in molecular biology, cancer, and viral infections, the potential of DDX21 as a therapeutic target becomes increasingly clear, underscoring its significance.

## 8. Future Directions

To address the gaps and challenges in DDX21 research, future studies should focus on high-resolution structural analysis to elucidate its molecular mechanisms, facilitating the design of specific inhibitors or activators. Investigating the context-dependent roles of DDX21 in various cancers and viral infections will provide deeper insights into its functional diversity. The development of targeted inhibitors or activators could offer new therapeutic strategies for cancer and viral diseases. Additionally, integrating computational biology, structural biology, and systems biology approaches will enhance our ability to study DDX21’s complex functions and interactions.

## Figures and Tables

**Table 1:** Regulation of DDX21 in different viruses.

Virus	Type	Regulatory Mechanism	Ref.
Hantaan virus	−ssRNA	DDX21 and DDX60 augment the interferon response.	[62]
Chum salmon reovirus	dsRNA	DDX21 upregulates type I interferon.	[63]
Spring viremia of carp virus	−ssRNA	DDX21 upregulates type I interferon.	[63]
SARS-CoV-2	ssRNA	The interaction between the Omicron N protein and human DDX21 may contribute to increased production of the anti-inflammatory cytokine IL-4.	[64]
Influenza A virus	−ssRNA	DDX21-TRIF-S100A9-TLR4-MyD88 signaling network is involved in regulating inflammation during infection.	[65]
		In the early stages, DDX21 binds to the viral polymerase PB1 to inhibit the replication of the virus. In the late stages, the NS protein overcomes this inhibition by binding to the N-terminal domain of DDX21, replacing PB1.	[66]
Human cytomegalovirus	dsDNA	DDX21 resolves R-loop to contribute to the transcription of late viral genes.	[67]
Porcine reproductive and respiratory syndrome virus	+ssRNA	DDX21 is translocated to the cytoplasm, stabilizing the expression of PRRSV nsp1α, nsp1β, and N proteins and inhibiting the host’s innate immune response.	[14]
Porcine circoviruses	circular ssDNA	The CTD of DDX21 bind the NoLS of the PCV4 Cap, targeting the PCV4 Cap to the nucleolus.	[11,21]
Human immunodeficiency virus type 1	Retrovirus +RNA	DDX21 can bind HIV rev proteins to regulate gene transcription late in infection.	[18,68]
Dengue virus	ssRNA	In the early stages, DDX21 is translocated from the nucleus to the cytoplasm to activate the innate immune response. In the late stages, DDX21 is degraded by the NS2B-NS3 protease complex of the virus, disrupting the innate immune response.	[69]
Foot-and-mouth disease virus	+ssRNA	The overexpression of DDX21 inhibited virus IRES-dependent translation and replication, and then FMDV and its non-structural proteins 2B, 2C, and 3Cpro degrade DDX21 through different pathways.	[70]

## Data Availability

No new data were created or analyzed in this study.
